# Adalimumab‐Induced Cutaneous Lupus Erythematosus: Cases Presenting With Malar Rash and Panniculitis

**DOI:** 10.1002/ccr3.70115

**Published:** 2025-01-14

**Authors:** Parvin Mansouri, Farahnaz Bidari‐Zerehpoosh, Nikoo Mozafari

**Affiliations:** ^1^ Medical Laser Research Centers, Academic Center of Education, Culture and Research Tehran University of Medical Sciences Tehran Iran; ^2^ Department of Pathology Loghman‐Hakim Hospital, Shahid Beheshti University of Medical Sciences Tehran Iran; ^3^ Skin Research Center Shahid Beheshti University of Medical Sciences Tehran Iran; ^4^ Department of Dermatology Loghman Hakim Hospital, Shahid Beheshti University of Medical Sciences Tehran Iran

**Keywords:** cutaneous lupus, lupus panniculitis, paradoxical reactions, psoriasis, tumor necrosis factor‐alpha (TNF‐α) inhibitors

## Abstract

Tumor necrosis factor‐alpha (TNF‐α) inhibitors are commonly used for management of various autoimmune disorders but can rarely cause isolated cutaneous lupus. This report presents two cases of cutaneous lupus erythematosus (LE) in women aged 38 and 61 after adalimumab treatment for psoriasis highlighting the importance of recognizing these paradoxical reactions for timely management.

## 1 Introduction

Tumor necrosis factor‐α (TNF‐α) inhibitors represent a class of biologic agents extensively utilized in the management of various autoimmune disorders [1]. This group encompasses infliximab, certolizumab pegol, etanercept, golimumab, and adalimumab, which are used for the treatment of rheumatoid arthritis (RA), juvenile idiopathic arthritis, psoriatic arthritis, ankylosing spondylitis, Crohn's disease, ulcerative colitis, plaque psoriasis hidradenitis suppurativa, and uveitis [1].

TNF‐ α inhibitors have unique structures that can induce immune responses, which may result in the development of paradoxical autoimmune phenomena, such as systemic lupus erythematosus (SLE) [2].

In addition to systemic adverse effects, anti‐TNF agents are now increasingly known to cause a wide range of cutaneous reactions [3]. One uncommon and seldom recognized side effect of treatment with anti‐TNF agents is the development of isolated cutaneous lupus [3].

Isolated cutaneous lupus is reported to primarily manifests in the forms of discoid [4] and subacute cutaneous lupus [5]. Instances of isolated cutaneous lupus presenting as acute cutaneous lupus (malar rash) [6] or isolated lupus panniculitis [7] associated with TNF‐α inhibitors are rare and infrequently documented.

Here, we present two cases of cutaneous lupus erythematosus (CLE) occurring in a 38‐year‐old and a 61‐year‐old woman following the use of adalimumab for the treatment of their psoriasis. These cases manifested as a malar rash and panniculitis, respectively.

## 2 Case One

### 2.1 Case History/Examination

A 38‐year‐old woman presented for evaluation of a rash on her face that began 2 months earlier. Initially, the patient was treated with mometasone and ketoconazole cream, with a diagnosis of seborrheic dermatitis. However, the rash did not respond to treatment and instead progressed, spreading to encompass her cheeks and interdigital spaces of her hands. The patient had a longstanding diagnosis of psoriatic arthritis and had not achieved a complete response to methotrexate. Approximately 1 year ago, she had been initiated on adalimumab (40 mg subcutaneous every 2 weeks).

**FIGURE 1:**
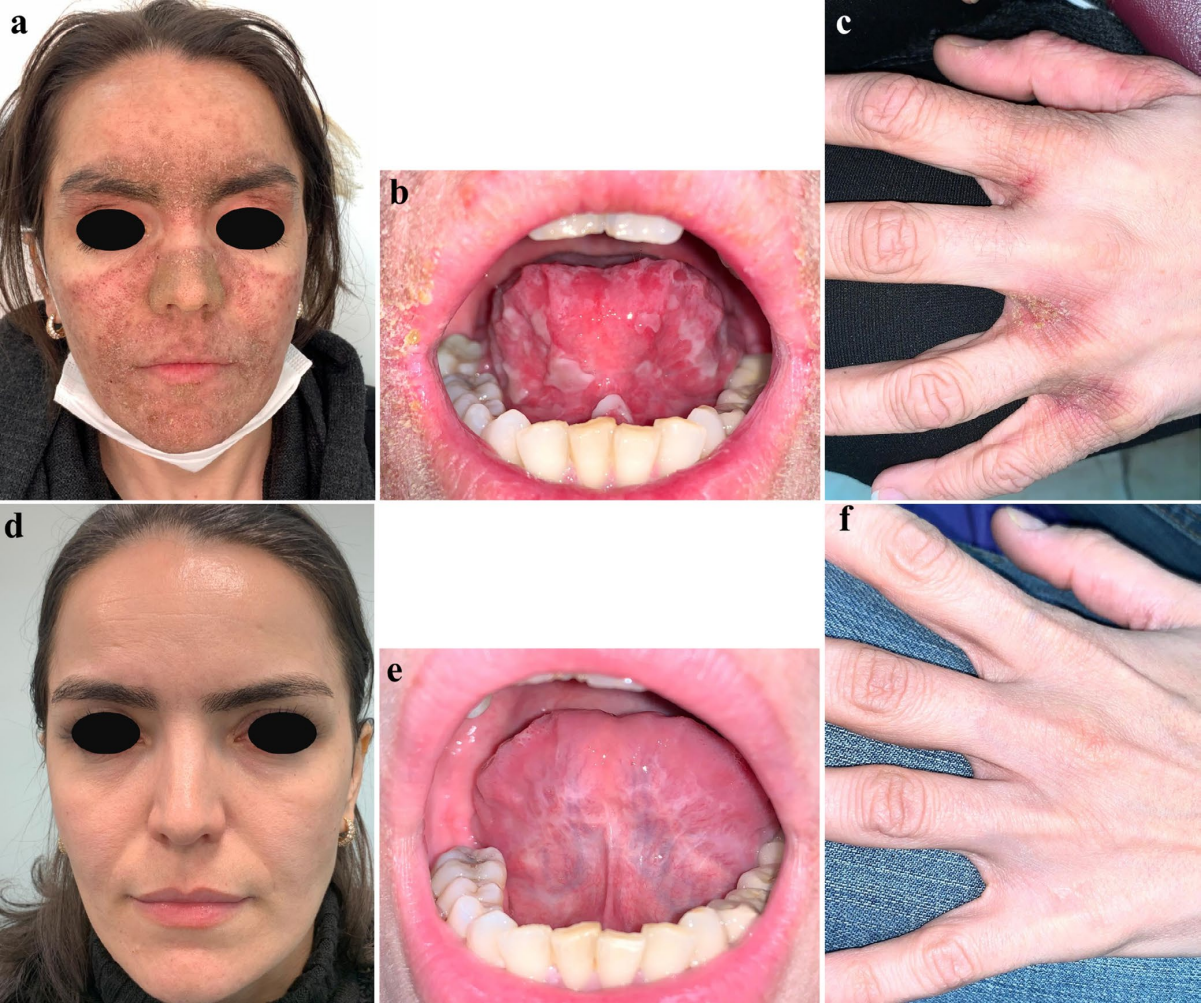
(a) Erythematous scaly plaques over the nose and malar cheeks. (b) Superficial erosions were observed on the tongue. (c) Interdigital erythema and scaling were also evident. (d–f) After treatment with hydroxychloroquine there was marked improvement in the mucocutaneous findings.

The physical examination revealed erythematous scaly plaques over the nose and malar cheeks. Interdigital erythema and scaling were also evident. Multiple scaly plaques were noted on the upper eyelids. Additionally, superficial erosions were observed on the tongue and buccal mucosa. No arthritis or other systemic symptoms were detected upon systemic physical examination. (Figure 1a–c).

### 2.2 Methods (Investigations and Treatment)

Laboratory investigations revealed a positive antinuclear antibody (ANA) titer of 1:160. However, anti‐ds‐DNA, anti‐RO, anti‐La, and anti‐CCP antibodies were within normal ranges. Additional studies, including C‐reactive protein, erythrocyte sedimentation rate, blood urea nitrogen, creatinine, aminotransaminases, urine analysis, and complete blood count, all yielded results within normal limits.

**FIGURE 2:**
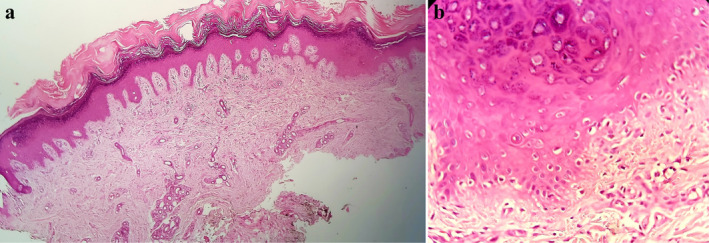
(a) Histopathologic examination revealed hyperkeratotic epidermis with Lymphocytic infiltration of papillary dermis (×10), (b) interface infiltration of small mature lymphocytes accompanied by vacuolar degeneration of basal layer (×40).

A skin biopsy from face revealed hyperkeratotic epidermis with lymphocytic infiltration of dermis. interface dermatitis was evident as infiltration of small mature lymphocytes accompanied by vacuolar degeneration of basal layer (Figure 2). Direct immunofluorescence of the lesional skin revealed deposits of IgM and IgG at the dermo‐epidermal junction with granular patterns, consistent with cutaneous lupus erythematosus (LE). Given the clinical and histopathologic findings, there was a specific concern for adalimumab‐induced cutaneous LE.

### 2.3 Conclusion and Results (Outcome and Follow‐Up)

Adalimumab was discontinued, and treatment was initiated with hydroxychloroquine at a dosage of 200 mg twice daily. Additionally, topical tacrolimus 0.1% ointment was applied twice daily for the facial rash. After 4 weeks, there was marked improvement in the cutaneous findings, and complete resolution of the rash was achieved within 3 months. (Figure 1d–f).

## 3 Case Two

### 3.1 Case History/Examination

**FIGURE 3:**
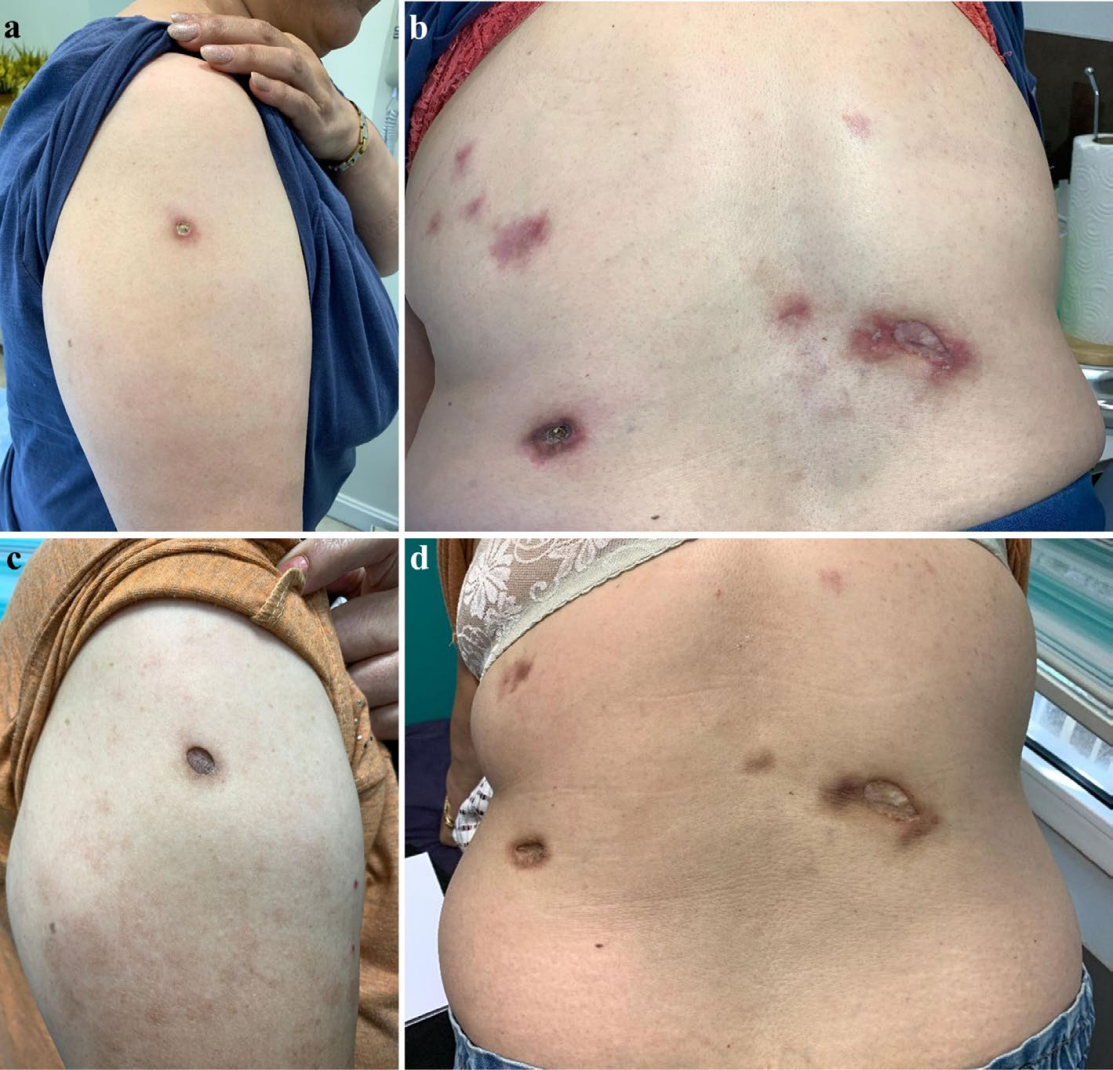
(a, b) Erythematous, tender and indurated subcutaneous plaques on the back and arm. (c, d) After discontinuation of adalimumab and treatment with thalidomide reduction in tenderness, erythema, and induration were observed.

A 61‐year‐old woman with cutaneous and arthritic psoriasis, initially controlled for 2 years with methotrexate (15 mg/weekly), faced concerns regarding long‐term use due to the risk of liver fibrosis. To taper methotrexate, adalimumab was introduced at a dose of 40 mg subcutaneously every 2 weeks. Nine months later, the patient developed erythematous, tender and indurated subcutaneous plaques on her back and arm. (Figure 3a,b).

No constitutional symptoms such as fever, malaise, or weight loss were noted. Additionally, there were no symptoms of arthritis in the form of joint tenderness, swelling, or effusion. No other significant examination findings were observed.

### 3.2 Methods (Investigations and Treatment)

Laboratory evaluation revealed an ANA titer of 1:160 with a homogeneous pattern. dsDNA antibodies were strongly positive, and complement components C3 and C4 were decreased. However, full blood count, immunoglobulins, erythrocyte sedimentation rate, C‐reactive protein, creatinine, urea, and electrolytes were within normal limits.

Skin biopsy showed lymphocytic infiltrations from the upper dermis to the deep dermis with perivascular and periadnexal involvement. lymphocytic panniculitis with hyaline necrosis of the subcutaneous fat were also observed. In direct immunofluorescence study positive IgM deposition in the dermoepidermal junction was observed.

### 3.3 Conclusion and Results (Outcome and Follow‐Up)

With diagnose of lupus panniculitis, adalimumab was discontinued, and methotrexate was reintroduced at a dose of 20 mg/week. Although the patient did not develop new lesions after stopping adalimumab, existing lesions did not regress. Subsequently, thalidomide 100 mg twice daily was initiated 4 months ago. While the lesions have not disappeared, there has been a reduction in tenderness, erythema, and induration. (Figure 3c,d).

## 4 Discussion

Lupus‐like paradoxical reactions (L‐PRs) induced by TNF‐α inhibitors are relatively uncommon but have been well documented [3]. The prevalence of anti‐TNF therapy‐induced Lupus‐like paradoxical reactions is rare, estimated to occur in approximately 0.5%–1% of treated patients [8]. The clinical presentation of Lupus‐like paradoxical reactions may vary widely, ranging from mild cutaneous manifestations, such as erythematous macules and papules, to more severe forms, including subacute cutaneous lupus erythematosus (SCLE) or SLE exacerbation [8].

The average time to onset of lupus‐like paradoxical reactions (L‐PRs) is approximately 14.6 months, although onset may occur in less than 1 month or extend beyond 6 years after initiation of TNF‐α inhibitor therapy [3].

The mechanism by which TNF‐α antagonists induce lupus‐like paradoxical reactions (L‐PRs) remains poorly understood. Some authors have suggested that adalimumab induces apoptosis, leading to the accumulation of nucleosomal antigens from apoptotic cells, which in turn triggers autoantibody production in susceptible individuals [9]. Additionally, it has been postulated that TNF‐α antagonists may impair T‐helper type 1 cytokine production and shift toward a Th2 immune response, which could be pathogenic through the production of autoantibodies [9]. Furthermore, the inhibition of TNF‐α blocks the induction of cytotoxic T‐lymphocytes, which normally function to suppress autoreactive B lymphocytes, thereby potentially contributing to humoral autoimmunity [9].

Another mechanism involves the interaction of TNF‐alpha inhibitors with interferon and plasmacytoid dendritic cells (pDCs) [10]. pDCs play a key role in the pathogenesis of cutaneous lupus erythematosus (CLE) by creating a connection between the innate and adaptive immune systems [11]. Type I interferons (IFN) are essential for the maturation and migration of plasmacytoid dendritic cells (pDCs) [11]. It is well‐established that type I IFN plays a central role in driving CLE [11]. TNF‐α is believed to suppress IFN‐α expression and act as an antagonist to the type I IFN pathway [10]. According to Palucka et al., patients receiving TNF‐α inhibitors showed increased transcription of IFN‐stimulated genes [12]. This mechanism can lead to an uncontrolled rise in IFN‐α levels, ultimately triggering lupus‐like reactions [10].

The primary approach in the treatment of anti‐TNF‐α lupus‐like paradoxical reactions is discontinuation of the causative agent [9]. Symptoms may resolve within 3 weeks–6 months after withdrawal of the implicated drug, and recurrence is uncommon thereafter [3]. However, in some cases, in addition to discontinuing anti‐TNF therapy, corticosteroids and immunosuppressive agents might be required to achieve full resolution of lupus symptoms [9].

In our report, in the case presenting with a malar rash, complete healing was achieved upon discontinuation of adalimumab and initiation of hydroxychloroquine. However, in the case presenting as panniculitis, although the progression of the lesions was halted, resolution has not been achieved even after thalidomide was initiated.

After resolution, the safety of re‐challenging patients who experienced paradoxical reactions to TNF‐alpha inhibitors with the same medication or another anti‐TNF‐alpha agent requires further investigation, as there is currently insufficient evidence to support this practice [9]. Reports focused on paradoxical psoriasis following TNF‐alpha inhibitor use indicate that in patients who experienced paradoxical psoriasis with one TNF‐alpha inhibitor, switching to another anti‐TNF‐α agent led to a recurrence of lesions in about half of the cases. Generally, in patients with moderate to severe paradoxical reactions, switching within the same class of drugs is not recommended. Instead, initiating therapy with a different drug class is advised [13, 14].

## 5 Conclusion

We describe two cases of patients who developed two distinct cutaneous presentations of LE after 9–12 months of initiating anti‐TNF therapy (adalimumab) for the treatment of psoriasis. Familiarity with the clinical features of paradoxical reactions from anti‐TNF‐α inhibitors may enhance the ability of healthcare workers to promptly recognize and manage this rare yet clinically significant complication. Early recognition remains a cornerstone in effective management.

## Author Contributions

**Parvin Mansouri:** conceptualization; data curation. **Farahnaz Bidari‐Zerehpoosh:** data curation. **Nikoo Mozafari:** conceptualization; data curation; writing – original draft; writing – review and editing.

## Disclosure

We declare that none of the authors listed on the manuscript are employed by a government agency that has a primary function other than research and/or education. And none of the authors are submitting this manuscript are as an official representative or on behalf of the government.data availability statement: the data presented in this study are available on request from the corresponding author.

## Consent

The two patients in this manuscript gave written informed consent for the publication of their case details.

## Conflicts of Interest

The authors declare no conflicts of interest.

## Data Availability

The data that support the findings of this study are available from the corresponding author upon reasonable request.
